# Experiences and Perceptions of Food Avoidance in Patients With Inflammatory Bowel Disease: A Qualitative Meta-Synthesis

**DOI:** 10.1097/jnr.0000000000000686

**Published:** 2025-08-01

**Authors:** Qingyu Wang, Sha Li, Junyi Gu, Jiefeng Yang, Jiali Chen, Hantian Cheng, Zheng Lin, Yang Lei

**Affiliations:** 1 Department of Cardiology, Affiliated People’s Hospital of Jiangsu University, Zhenjiang, China; 2 School of Nursing, Nanjing Medical University, Nanjing, China; 3 Health School, Shanghai University of Medicine & Health Sciences, Shanghai, China; 4 Department of Gastroenterology, The First Affiliated Hospital of Nanjing Medical University, Nanjing, China; 5 Department of Nursing, The First Affiliated Hospital of Nanjing Medical University, Nanjing, China • †Contributed equally

**Keywords:** inflammatory bowel disease, food avoidance, qualitative research, meta-synthesis

## Abstract

**Background:**

Food avoidance, common in patients with inflammatory bowel disease (IBD), impacts their recovery and psychological health. However, limited insight is provided in the literature regarding the food avoidance experiences and perceptions of patients. A systematic qualitative synthesis exploring these experiences and perceptions may be expected to enhance scholarly understanding of their implications for IBD.

**Purpose:**

This study was developed to review and characterize existing qualitative studies on food avoidance in patients with IBD and to use relevant data from previous studies to guide and optimize diet management strategies for patients.

**Methods:**

Eight databases (PubMed, Embase, Cochrane Library, Web of Science, EBSCO, and three Chinese databases) were searched to identify qualitative studies on the food avoidance experiences and perspectives of patients with IBD. Critical Appraisal Skills Program (CASP) Qualitative Checklists were applied to appraise the included studies, and a meta-synthesis approach was used to analyze the data. The findings and quotations from the studies were recharacterized into new themes and categories using inductive thematic synthesis and reciprocal interpretation.

**Results:**

Of the 1,224 studies retrieved, 19 were included in this meta-synthesis. The experiences and feelings of food avoidance in patients with IBD included the four categories of Coping Strategies, Disruption of Life and Mood, Needs and Expectations, and Social Alienation, from which the following eleven themes were derived: (a) motivations and perspectives, (b) safe recipes updated in failure, (c) positive impact, (d) reshaping life planning and increasing life stress, (e) negative emotional challenges, (f) the role of family and friends in dietary management, (g) workplace support deficiencies, (h) lack of professional dietary guidelines, (i) alienation from intimacy, (j) alienation of culinary culture, and (k) social distancing.

**Conclusions/Implications for Practice:**

The findings of this study highlight the complexities that underlie food avoidance behaviors in people with IBD and reveal the challenges faced by patients in managing their diet and emotions. The importance of personalized dietary guidance based on food avoidance, negative emotion de-escalation, and strong social support for disease management is highlighted.

## Introduction

Inflammatory bowel disease (IBD), encompassing ulcerative colitis (UC) and Crohn’s disease (CD), represents a spectrum of chronic, idiopathic gastrointestinal disorders. Symptoms include diarrhea, abdominal pain, mucous pus, blood stains, and postural rigor, among others. The number of IBD cases worldwide number over 4.9 million, and the prevalence of this disease continues to rise (Wang et al., 2023). IBD has an unclear etiology and often relapses, with no cure available now. Food affects the immune function, regulates gene-environment interactions, and alters the gut microbiome, all of which contribute to the development and progression of IBD (Adolph & Zhang, 2022).

Consequently, diet management has emerged as a crucial behavioral intervention for people with IBD and is a focus for both patients and researchers (Fitzpatrick et al., 2022; Geldof et al., 2020). Contemporary strategies in IBD care prioritize identifying and rectifying malnutrition and dietary imbalances as a foundational aspect of patient management (Fitzpatrick et al., 2022). Nonetheless, food avoidance is common among patients with IBD (Bonsack et al., 2023; Marsh et al., 2019).

Food avoidance refers to the intentional avoidance of certain types or categories of food (Godny & Dotan, 2023). While patients adopt food avoidance to reduce symptoms or prevent disease progression (Day et al., 2021), this strategy is detrimental to disease repair and may lead to malnutrition, eating disorders, and reduced quality of life (Bonsack et al., 2023; Casanova et al., 2017). The results of related research, primarily cross-sectional, have yet to establish causal relationships between diet and IBD, with the lack of specific and clear dietary guidelines complicating patient decision-making (Bischoff et al., 2023). This challenge is exacerbated by individual variability in food tolerance, cultural dietary norms, and other factors (Alexakis et al., 2015). Reports in the literature regarding the results of quantitative analyses show that, on average, patients with IBD avoid 6.3±3.7 food types, with over 86% avoiding certain foods when the disease is active and 74% during remission (Casanova et al., 2017; Marsh et al., 2019; Wang et al., 2022). Foods that may reduce inflammation and prevent secondary disease are frequently mistakenly excluded from this type of self-selection food avoidance (Bonsack et al., 2023; Godala et al., 2023).

Significant research has already been conducted to investigate how food avoidance affects the daily lives and psychological well-being of patients with IBD, with numerous qualitative studies delving into their diet management experiences. While qualitative studies cannot reflect the experience of this population definitively, systematic reviews can elucidate the when, how, and why behind food avoidance behaviors in patients with IBD (Zimmer, 2006). In 2022, a review synthesized 14 qualitative studies on the impact of diet on the quality of life of patients suffering from IBD. However, that study did not address the underlying motivations, needs, or characteristics of those practicing food avoidance (Palamenghi et al., 2022), which is crucial in enhancing patient dietary practices and clinical guidance. In this study, the domestic and international qualitative research on this issue is systematically evaluated, with findings from the Enhancing Transparency in Reporting the Synthesis of Qualitative Research statement (Tong et al., 2012) synthesized. The objective of this study is to provide a nuanced interpretation of the food avoidance experiences of patients with IBD and to identify current gaps in dietary management to inform future guidelines.

## Methods

### Research Design

A systematic review and qualitative meta-synthesis (PROSPERO CRD42023406454) was conducted and findings were reported in line with the Preferred Reporting Items for Systematic Reviews and Meta-Analyses and Enhancing Transparency in Reporting the Synthesis of Qualitative Research statement (Moher et al., 2009; Tong et al., 2012). Key themes and categories were extracted from the included studies using the meta-synthesis strategy (Zimmer, 2006).

### Inclusion and Exclusion Criteria

SPIDER (Sample, Phenomenon of Interest, Design, Evaluation, and Research Type; Cooke et al., 2012) is a widely used tool for formulating qualitative research questions. Based on the objectives, the studies were chosen, and inclusion criteria were created using the SPIDER tool (Table 1).

**Table 1:** Inclusion and Exclusion Criteria

Inclusion Criteria (SPIDER)		Exclusion Criteria
S:	Patients with a definite medical diagnosis of IBD and no co-existing disease affecting the study results showed good expression ability	Patients undergoing total enteral or parenteral nutrition • No review or protocol or animal study • Qualitative data cannot be extracted from quantitative studies • Nonpublished journal articles
PI:	Experiences (or perspective, attitude, knowledge, perception, etc.) of IBD living with food avoidance	
D:	Interview, focus group, group discussion, questionnaire	
E:	Thematic analysis, content analysis, ethnography, narrative, descriptive, etc.	
R:	Qualitative research or mixed methods	

*Note.* S = sample, PI = phenomenon of interest, D = design, E = evaluation, R = research type; IBD = inflammatory bowel disease.

### Search Strategy

First, to reduce publication and outcome reporting biases, all high-quality data on dietary avoidance among patients with IBD were included (Chiocchia et al., 2021). Next, two independent researchers conducted a thorough literature search and review. When topic-related conference papers or unpublished studies were found, the researchers requested additional data from the authors.

Searches were conducted in eight electronic databases: PubMed, Embase, Cochrane Library, Web of Science, EBSCO, China National Knowledge Infrastructure, Wanfang, and China Science and Technology Journal Database (VIP) for the period extending from their inception to March 23, 2023. In addition, the reference sections of the identified studies were hand searched for other potentially relevant studies. After conducting an initial limited search on PubMed, a comprehensive search strategy was developed using the SPIDER tool for qualitative synthesis. The Chinese keywords used were similar to the English keywords used. The search strategy formulated based on the SPIDER tool was (inflammatory bowel disease* OR colitis* OR crohn*) AND (nutrition* OR nutrient* OR food* OR diet* OR eat* OR meal*) AND (avoid* OR restrict* OR limit* OR manage* OR control*“) AND (qualitative OR grounded theory OR ethnology OR ethnography OR focus group OR observation OR interview OR phenomenolog* OR action research OR narrative analysis OR context analysis OR thematic analysis).

### Study Selection

Two authors independently utilized NoteExpress V3.0, a bibliographic application, to organize their selected publications. The software then imported these publications, eliminated duplicates, and screened based on titles and abstracts. The full-text papers were then downloaded and thoroughly vetted in terms of the inclusion and exclusion criteria. A third author resolved any disagreements or discrepancies with the first two authors through discussion.

### Quality Appraisal

The selected articles were rigorously checked for quality to ensure research reliability and validity. In addition, the researchers evaluated risk bias and its potential impact on the meta-synthesis. Based on the study design criteria, the Joanna Briggs Institute (JBI) was chosen for this research (Lockwood et al., 2020).

Two researchers independently analyzed the JBI, and conflicting results were discussed and resolved by the group. The JBI comprises 10 items, and each item is assigned one of four possible descriptors: “yes,” “no,” “unclear,” and “not applicable.” The total number of “yes” items is used to grade the reliability and validity of articles, with 10 “yes” items resulting in an “A” grade (low risk of bias and high study quality), 6–9 items resulting in a “B” grade (moderate risk of bias and medium study quality), and <5 items resulting in a “C” grade (high risk of bias, study design issues, and low quality; Boehm et al., 2021). Only A and B-grade studies were analyzed in this study to uphold meta-synthesis quality and scientific validity.

### Data Extraction

Concurrent with the assessment of study quality, data extraction was meticulously performed. IBD food avoidance data were extracted from the three included studies that addressed IBD and patients with irritable bowel syndrome. Two of the included studies examined patient self-management and lived experiences with regard to food avoidance. Beyond food avoidance data, the first author, publication year, country, study aim (s) or issue (s), study design, study setting, recruitment strategy, sample size (IBD type, gender), age of sample, time of IBD diagnosis, data collection methods, data analysis strategy, ethical issues, data saturation, researcher–participant relationship, and statement of findings were also collected.

### Data Analysis and Synthesis

Data analysis and synthesis were conducted using thematic analysis that adhered to a six-phase process outlined in prior research (Braun & Clarke, 2006). First, familiarization involved reading and annotating IBD patient transcripts, using NVivo12. Second, initial code generation entailed systematically coding relevant text segments to identify key features. Third, theme generation involved grouping codes to form themes. Fourth, theme review involves iterative reading, discussion, and reflection, during which understanding of the data is gradually deepened and codes and themes are continuously optimized. Fifth, themes were defined and named with clarity, accompanied by analytic descriptions to reflect their core meanings. Sixth, report preparation. Two of the authors who were trained in evidence-based methodology and qualitative research completed and cross-checked each part independently, with disagreements resolved through mutual discussion or in consultation with the third author. All of the authors discussed the thematic framework and analysis procedures. Everyone on the team had prior clinical and IBD research experience.

## Results

### Literature Retrieval

**Figure 1:**
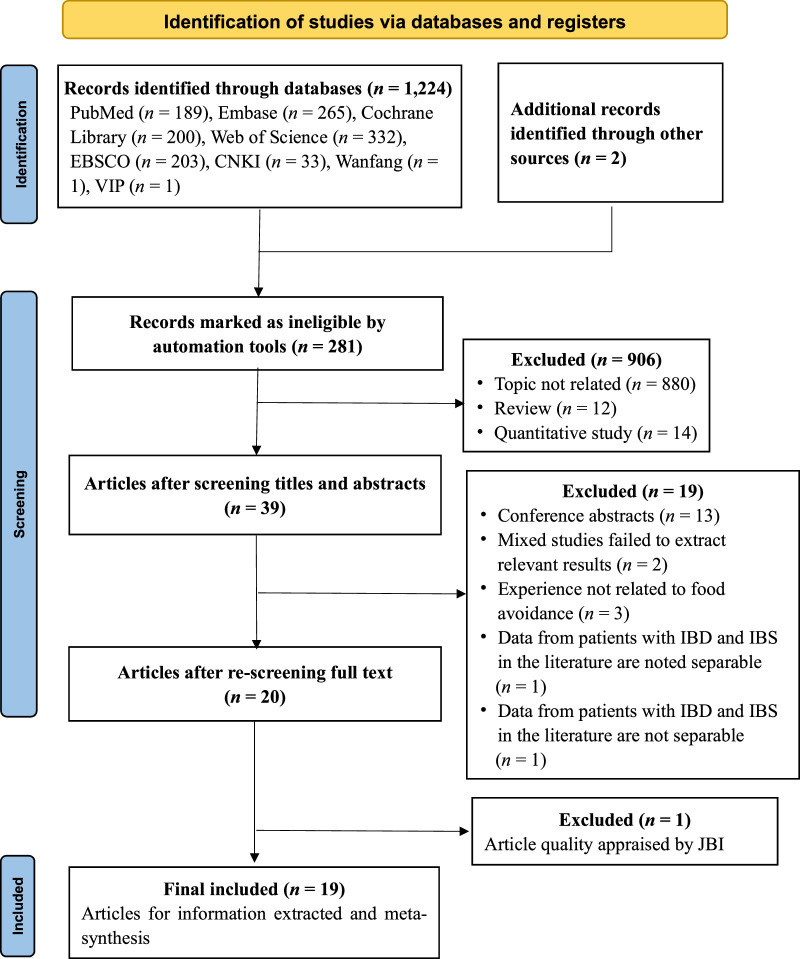
Preferred Reporting Items for Systematic Reviews and Meta-Analyses—Study Selection Flowchart *Note*. CNKI = China National Knowledge Infrastructure; VIP = China Science and Technology Journal Database; IBD = inflammatory bowel disease; JBI = Joanna Briggs Institute.

The process used to select the articles is shown in Figure 1. The one article in Spanish that was selected was translated into English by translators and checked for translation accuracy.

### Quality Appraisal

The final integrated appraisal table (after author discussion) is presented in Table 2. One article was excluded from this comprehensive review due to ambiguities in the recruitment strategy used, questionable interpretation of the results, and inadequate clarity regarding the cultural/theoretical perspective of the researcher.

**Table 2:** Study Quality Appraisal Details Using the JBI Tool

Study	①	②	③	④	⑤	⑥	⑦	⑧	⑨	⑩	Overall Appraisal	Result
Matinez-Riera et al., 2022	Yes	Yes	Yes	Yes	Yes	Yes	Yes	Yes	Yes	Yes	A	Included
Rines et al., 2022	Yes	Yes	Yes	Yes	Yes	Yes	Yes	Yes	Yes	Yes	A	Included
Rogers et al., 2021	Yes	Yes	Yes	Yes	Yes	No	Unclear	Unclear	Yes	Yes	B	Included
Barriteau Siiri et al., 2022	Yes	Yes	Yes	Yes	Yes	Yes	Yes	Yes	Yes	Yes	A	Included
Scott et al., 2021	Yes	Yes	Yes	Yes	Yes	Yes	Yes	Yes	Yes	Yes	A	Included
Czuber-Dochan et al., 2020	Yes	Yes	Yes	Yes	Yes	Yes	Yes	Yes	Yes	Yes	A	Included
Nowlin et al., 2021	Yes	Yes	Yes	Yes	Yes	No	Unclear	Yes	Yes	Yes	B	Included
Chuong et al., 2019	Yes	Yes	Yes	Yes	Yes	No	Unclear	Unclear	Yes	Yes	B	Included
Chen et al., 2017	Yes	Yes	Yes	Yes	Yes	Yes	Unclear	Unclear	Yes	Yes	B	Included
Lovén Wickman et al., 2016	Yes	Yes	Yes	Yes	Yes	Unclear	Unclear	Unclear	Yes	Yes	B	Included
Alexakis et al., 2015	Yes	Yes	Yes	Yes	Yes	Yes	Yes	Yes	Yes	Yes	A	Included
García-Sanjuán et al., 2015	Yes	Yes	Yes	Yes	Yes	No	Unclear	Yes	Yes	Yes	B	Included
Palant et al., 2015	Yes	Yes	Yes	Yes	Yes	Unclear	Yes	Yes	Yes	Yes	B	Included
Skrautvol & Nåden, 2015	Yes	Yes	Yes	Yes	Yes	Unclear	Yes	Yes	Yes	Yes	B	Included
Sykes et al., 2015	Yes	Yes	Yes	Yes	Yes	Yes	Yes	Unclear	Yes	Yes	B	Included
Zhou et al., 2014	Yes	Yes	Yes	Yes	Yes	Unclear	Unclear	Yes	Yes	Yes	B	Included
Holst et al., 2014	Unclear	Yes	Yes	No	No	No	Yes	Yes	Unclear	Yes	C	Excluded
Fletcher et al., 2008	Yes	Yes	Yes	Yes	Yes	No	Unclear	Unclear	Yes	Yes	B	Included
Jamieson et al., 2007	Yes	Yes	Yes	Yes	Yes	No	No	Unclear	Unclear	Yes	B	Included
Fletcher & Schneider, 2006	Yes	Yes	Yes	Yes	Yes	No	No	Unclear	No	Yes	B	Included

*Note.* ① Is there congruity between the stated philosophical perspective and the research methodology?; ② Is there congruity between the research methodology and the research question or objectives?; ③ Is there congruity between the research methodology and the methods used to collect data?; ④ Is there congruity between the research methodology and the representation and analysis of data?; ⑤ Is there congruity between the research methodology and the interpretation of results?; ⑥ Is there a statement locating the researcher culturally or theoretically?; ⑦ Is the influence of the researcher on the research, and vice versa, addressed?; ⑧Are participants, and their voices, adequately represented?; ⑨ Is the research ethical according to current criteria or, for recent studies, and is there evidence of ethical approval by an appropriate body?; ⑩ Do the conclusions drawn in the research report flow from the analysis, or interpretation, of the data?

### Study Description

Because most of the articles included in this study address food avoidance from the perspective of patient dietary experiences, the focus of this study, as shown in Table 3, was on extracting, reintegrating, and analyzing data related to the experiences and feelings of food avoidance. The 19 included articles were published between 2006 and 2022 and were conducted in nine different countries. The research designs used included descriptive qualitative research, exploratory qualitative research, ethnography, narrative inquiry, explorative hermeneutic research, naturalistic inquiry, and phenomenology. The focus of all of the included studies was on participants’ subjective experiences, with recruitment strategies including purposive and snowball sampling, and participants were predominantly young and middle-aged adults, with seven (36.8%) of the studies also including older adults and children (individuals aged ≥65 years or <18 years). All of the participants had experienced IBD for over four months. Data collection was primarily done using semistructured interviews, with two (10.5%) of the studies using focus groups and narrative interviews. Thematic, content, or grounded theory were the primary modes used in data analysis. Only one (5.3%) study did not report receiving the approval of either a Research Ethics Committee or institutional review board. Nine (47.4%) of the included studies discussed data saturation, while six (31.6%) critically examined the researcher–participant relationship. All of the studies stated their findings clearly.

**Table 3:** Data Extraction

Study/Country	Study Aim (s)/Issue (s)	Study Design Study Setting, Recruitment Strategy	Sample Size (IBD Type, Gender), Age of Sample, Time of Diagnosis of IBD	Philosophical Foundation, Data Collection Method, Data Analysis Strategy, Ethical Issues	Data Saturation/Relationship Between Researcher and Participants/Statement of Findings	The Primary Outcome
Matinez-Riera et al., 2022/ Spain	To establish how eating during the working day affects people diagnosed with IBD, and the barriers and feeding strategies they have to employ to reconcile work demands	Online meetings Purposive and snowball sampling methods	7 participants (3UC/4CD, 4M/3F) Aged between 26 and 53 years More than 5 years	Constructivist paradigm Focus group discussions (Nominal Group and Focus Groups) Thematic analysis Ethics approval from the Research Ethics Committee	No reported, clear statement of findings Clear statement of findings	1. Management of food during the working day. 2. Searching for strategies to live with the disease. 3. The importance of visibility and support.
Rines et al., 2022/ Canada	To explore the psychosocial experiences that young adults with IBD have with food via a qualitative patient-led research process	Online meetings Snowball sampling	9 participants (4UC/6CD, 3M/6F) Ages between 18 and 35 years Ranged between 3 and 16 years	Semistructured interviews and focus groups Thematic analysis Ethics approval from the Research Ethics Committee	Thematic saturation Relationship stated Clear statement of findings	1. Experimenting with Food. 2. Evolution Over Time. 3. Diet Changes are Emotional. 4. The role of stigma.
Rogers et al., 2021/United States	To investigate diet and nutrition management on IBD patients as biform work, identify components of articulation work, and provide guidance on how to design consumer health information technology	Qualitative research Over the phone or on a video-chat platform Convenience sampling	21 participants (21CD, 5M/16F) Ages between 20 and 64 years Average of 12 years	Semi-structured interviews Conventional content analysis Ethics approval from the Institutional Review Board and informed consent	Data saturation Relationship stated, Clear statement of findings	1. Physical management. 2. Emotional management. 3. Information management. 4. Technology-enabled management.
Barriteau Siiri et al., 2022/ Norway	To explores how patients with IBD experience dietary guidance provided by different health professionals	Descriptive qualitative research, Convenient location for interviews and observation recording Purposive sampling	10 participants (4CD/6UC,1M/9F) Ages between 26 and 57 years More than 6 months	Semi-structured interviews Thematic analysis Ethics approval from the research center for data & informed consent	Thematic saturation Relationship stated Clear statement of findings	1. More need for dietary guidance. 2. Experiences of receiving dietary guidance from a registered dietitian. 3. Experiences with dietary guidance from other health professionals.
Scott et al., 2021/United States	To explore the beliefs and experiences of African Americans with IBD and coping in the context of their culture	Ethnography, In a private office or in a private conference room Snowball sampling	12 participants (9CD/4UC, 5M/7F) Ages between 25 and 68 years More than 3 years	Field work, participant observation, semistructured interviews, Ethics approval from the Institutional Review Board and informed consent	Data saturation, No reported Clear statement of findings	1. Spending time living in the bathroom. 2. Time and food restricted eating practices and cultural food avoidance. 3. Dealing with chronic stress and perceived racial injustice. 4. The practice of seclusion to manage bathroom urgency and emotions of fear, anxiety, and embarrassment.
Czuber-Dochan et al., 2020/United States	To explore people with IBD to explore the perceptions and psychosocial impacts of food, nutrition, eating and drinking	Phenomenology, The location is chosen by the patient Purposive sampling	28 participants (16CD/12UC, 13M/15F) Ages between 25 and 68 years Duration of diagnosis means 10 years	Semistructured interviews, Thematic analysis, Ethics approval from the National Research Ethics Committee & informed consent	Thematic saturation Relationship stated Clear statement of findings	1. Personal experience of relationship between IBD and food. 2. Managing diet to control IBD and its symptoms. 3. Impact of food-related issues on everyday life. 4. Acceptance and normalization of food and its impact in IBD. 5. Sources of information and support.
Nowlin et al., 2021/United States	To explore patients' beliefs, attitudes, and behaviors around medical treatments and medication therapies in IBD	Qualitative research No reported Purposive sampling,	16 participants (3CD/13UC, 5M/11F) Ages between 21 and 59 years Ranged from 5 months to 26 years	Semistructured interviews, Thematic analysis Ethics approval from the Institutional Review Board	Thematic saturation, Relationship stated Clear statement of findings	1. Evolving attempts at controlling symptoms through diet. 2. Beliefs about how food affects IBD. 3. Perceptive eating.
Chuong et al., 2019/ Canada	To explore how children and adolescents with IBD and their parents coped with the illness through food and diet in their daily lives	Exploratory qualitative research, Interviewed at clinic or by phone Purposive sampling	28 participants (23CD/5UC, 16M/12F) Ages between 9 and 17 years Ranged from 4 months to 11 years	Semi-structured interviews Thematic analysis Ethics approval from the Research Ethics Boards at The Children’s Hospital and the University	No reported No reported Clear statement of findings	1. Food avoidance and moderation. 2. Following a specific diet. 3. Healthy eating. 4 .Family food practices.
Chen et al., 2017/ China	To explore the knowledge, attitude and practice of dairy intake in patients with IBD	Descriptive qualitative research, The location is chosen by the patient Purposive sampling	21 participants (11CD/10UC,11M/10F) Ages between 22 and 43 years Ranged from 6 to 164 months	Semi-structured interviews Content analysis Ethics approval from the University’s Ethics Committee & informed consent	No reported No reported Clear statement of findings	1. The knowledge of dairy intake. 2. The attitude of dairy intake. 3. The practice of dairy intake.
Lovén Wickman et al., 2016/Sweden	To explore self-care among patients with IBD	Descriptive qualitative research, The location and format is chosen by the patient Purposive sampling	20 participants (12CD/8UC, 10M/10F) Ages between 25 and 66 years Ranged from 1 to 46 years	Semi-structured interviews Conventional content analysis Ethics approval from Regional Ethical Review Board & informed consent	No reported Relationship stated, Clear statement of findings	1. Symptom recognition. 2. Handling of symptoms. 3. Planning life. 4. Seeking new options.
Alexakis et al., 2015/UK	To identify and characterize the experiences and difficulties faced by young IBD patients from black and minority ethnic communities	Qualitative research The location and format is chosen by the patient Purposive sampling	20 participants (13CD/6UC/1 Other, 13M/7F) Ages between 16 and 24 years No reported	Semi-structured interviews Thematic analysis Ethics approval from the Research Ethics Committee &and informed consent	Credibility, dependability and confirmability Thematic saturation, Thematic saturation Relationship stated Clear statement of findings	1. Culture and religion. 2. Parents, families and the wider community. 3. Education. 4. Health care services and support.
García-Sanjuán et al., 2015/Spain	To explore the experience of IBD patients affected in relation to food intake	Ethnography, In participants’ homes Snowball sampling	19 participants (19CD, 7M/12F) Ages between 23 and 84 years Ranged from 4 to 70 years	Semi-structured interviews Thematic analysis Ethics approval from a Research Ethics Committee & informed consent	Data saturation, Thematic saturation No reported Clear statement of findings	1. Beliefs about nutrition and CD. 2. Changing eating habits. 3. Finding information about food and CD. 4. The role of professionals. 5. Self-management.
Palant et al., 2015/Germany	To understand from a patient perspective the significance of food, eating, and nutrition, including the role of food in the social life of patients with IBD	Narrative inquiry, In the patient’s home or hospital department Theoretical and Maximum variation sampling	44 participants (25CD/15UC/2 Other, 21M/23F) Ages between 17 and 79 years No reported	Narrative interviews Grounded theory Ethics approval from the local ethics committee	Theoretical saturation Relationship stated Clear statement of findings	1. Managing uncertainty. 2. Eating: between craving and aversion. 3. Being different. 4. Professional help as a further source of uncertainty.
Skrautvol & Nåden, 2015/Norway	To explore how young adult people living with IBD experienced that knowledge about their body symptoms and their food intake could promote recovery from their diagnosed disease	Explorative hermeneutic, research At the researcher’s office Purposive sampling	13 participants (7CD/6UC, 3M/10F) Ages between 18 and 45 years Ranged from 1 to 12 years	Semi-structured interviews, Kvale and Brinkmann’s 3 levels hermeneutic approach Ethics approval from Regional Ethical Review Board & informed consent	Data saturation No reported Clear statement of findings	1. Confidence with symptoms of disease as a source of recovery. 2. Nutritional recovery in different stages of IBD.
Sykes et al., 2015/Canada	To examine the lived experiences of women with IBD, by specifically exploring their management of their illness after diagnosis	Heuristic phenomenology No reported Purposive sampling	8 participants (6CD/2UC, 8F) Ages between 32 and 50 years More than 2 years	Semistructured interviews, background questionnaire Content analysis Ethics approval	No reported Relationship stated Clear statement of findings	1. Changes in dietary behaviors. 2. Adjusting the daily routine and. 3. Management through medication.
Zhou et al., 2014/China	To explore the dietary experiences of Chinese patients with IBD	Naturalistic inquiry, In a quiet office next to the hospital Purposive sampling	17 participants (12CD/5UC, 10M/7F) Ages between 18 and 57 years Ranged from 6 months to 13 years	In-depth interviews Thematic analysis Ethics approval from hospital ethics committee and informed consent	Thematic saturation No reported Clear statement of findings	1. Seeking dietary information. 2. Testing out. 3. Modifying diet. 4. Barriers to diet modification. 5 .Fear and stress.
Fletcher et al., 2008/Canada	To explore the adverse behaviors that women diagnosed with the GI disorders IBD and/or IBS engaged in with respect to the consumption of food, beverages, and medications	Phenomenology, No reported No reported	3 participants (2CD/2UC, 12M/13F) Ages between 18 and 23 years Can’t tell	Semistructured interviews, background questionnaires, and food diaries Critical content analysis Ethics approval from the University’s Ethics Committee	No reported No reported Clear statement of findings	1. Adverse behaviors.
Jamieson et al., 2007/Canada	To explore preillness and postdiagnosis dietary patterns of participants, with an emphasis on the changes, if any, that participants have made to their diet following diagnosis with IBD or IBS	Phenomenology, location that was convenient for participants Can’t tell	3 participants (2CD/2UC, 12M/13F) Ages between 18 and 23 years No reported	Semistructured interviews, background questionnaires, and food diaries No reported Informed consent from the participants	No reported No reported Clear statement of findings	1. Determining one’s diet.
Fletcher et al., 2008/Canada	To explore the relationship between food and IBD or IBS	Phenomenology, No reported Poster advertising	3 participants (3UC, 13F) Ages between 19 and 22 years Can’t tell	Semistructured interviews and background questionnaires, food diaries Critical content analysis No reported	No reported No reported Clear statement of findings	1. The consumption of food.

*Note.* IBD = inflammatory bowel disease; CD = Crohn’s disease; UC = ulcerative colitis; IBS = irritable bowel syndrome; M = male; F = female.

### Meta-Synthesis

Four themes emerged from the repeated reading and analysis of the 19 included studies. A summary of the results is presented in Table 4.

**Table 4:** New Themes and Categories From 19 Included Articles

Category	Theme	Theme Explanation
Coping strategies	1. Motivations and perspectives	Reasons why patients use food avoidance as a coping strategy
	2. Safe recipes updated in failure	Patients’ experiences of using food avoidance to cope with illness
	3. Positive impact	Patients use food avoidance to cope with the positive effects of the disease.
Disruption of life and mood	4. Reshaping life planning and increasing life stress	Patient’s experience of food avoidance in daily life
	5. Negative emotional challenges	Patients’ emotional damage from food avoidance
Needs and expectations	6. The role of family and friends in dietary management	Patients’ need for dietary support provided by family and friends
	7. Workplace support deficiencies	Patients’ need for dietary support provided by the Workplace
	8. Lack of professional dietary guidelines	Patients’ need for dietary support provided by the medical environment
Social alienation	9. Alienation from intimacy	Food avoidance causes social alienation from intimate relationship
	10. Alienation from Culinary Culture	Food avoidance causes social alienation from the food culture
	11. Social distancing	Food avoidance causes Patient social isolation

### Category 1. Coping Strategies

#### Theme 1. Motivations and Perspectives

This theme centers on the dietary management efforts of these patients, focusing mainly on food avoidance and patient perceptions. The main findings related to this theme include (a) following dietary advice without the prompting of negative prior experiences, (b) consuming certain foods makes symptoms worse during flare-ups, (c) avoiding foods believed to increase the intestinal burden, and (d) exhibiting individual variations in food avoidance practices.

Analysis of participant quotations from 11 of the included studies revealed three primary motivations for food avoidance in patients with IBD: blind adherence to dietary advice (received from medical professionals, peers, or the internet; Chen et al., 2017; Zhou et al., 2014), a belief that certain foods exacerbate flare-up symptoms (Chuong et al., 2019; Czuber-Dochan et al., 2020; Palant et al., 2015; Sykes et al., 2015), and avoidance of foods thought to worsen inflammatory responses or intestinal damage, even during symptom-free periods (Chen et al., 2017; Chuong et al., 2019; Czuber-Dochan et al., 2020; García-Sanjuán et al., 2015; Nowlin et al., 2021; Palant et al., 2015; Skrautvol & Nåden, 2015; Sykes et al., 2015; Zhou et al., 2014). For example, one participant in an included study reported discontinuing milk consumption based on medical advice despite experiencing no prior adverse reactions (Zhou et al., 2014). Another described restricting their diet to basic soup during flare-ups and avoiding raw vegetables and other irritants (Sykes et al., 2015). Still another described one participant who regularly avoided fruits, considering them to be harmful (Zhou et al., 2014).

In addition, the included studies support the presence of individual variance in food avoidance practices (Chen et al., 2017; Czuber-Dochan et al., 2020; Nowlin et al., 2021; Palant et al., 2015; Zhou et al., 2014). Due to the inherent variability in their normal diet, one participant stressed the importance of personal experimentation to identify foods that were tolerable (Palant et al., 2015).

#### Theme 2: Safe Recipes Updated in Failure

This theme encapsulates the efforts of the participants to identify “harmful foods” using a trial-and-error approach, promoting a dynamic process of food avoidance. Despite adherence to “safe” foods, patients often experienced symptoms, necessitating continual adjustments to their diets. The original findings are (a) developing food avoidance strategies after dietary trial failures and (b) experiencing symptom relapse despite adhering to “safe foods.”

The majority of the participants reported avoiding foods that had triggered symptoms previously during dietary trials (Chen et al., 2017; Czuber-Dochan et al., 2020; Fletcher et al., 2008; Fletcher & Schneider, 2006; García-Sanjuán et al., 2015; Jamieson et al., 2007; Palant et al., 2015; Rines et al., 2022; Rogers et al., 2021; Scott et al., 2021; Sykes et al., 2015; Zhou et al., 2014). However, many still faced relapses, even while following a “safe food” diet (Czuber-Dochan et al., 2020; Fletcher & Schneider, 2006; Palant et al., 2015; Rines et al., 2022; Rogers et al., 2021; Zhou et al., 2014). A notable pattern emerged in which patients expanded their lists of avoided foods progressively as foods initially deemed “safe” subsequently became “triggers.” One participant described this evolutionary process, relating that each flare-up led to the discovery of a new intolerable food (Rines et al., 2022).

#### Theme 3. Positive Impact

This theme describes the positive effects of food avoidance on patients with IBD. The original findings are (a) food avoidance promotes healthier eating choices; (b) food avoidance fits in with the needs of work life; and (c) disease perception improves food avoidance.

The majority of the participants in the included studies reported altering their diet in response to IBD, primarily by excluding certain foods, and gravitating toward healthier options (Alexakis et al., 2015; Chuong et al., 2019; Czuber-Dochan et al., 2020; Fletcher & Schneider, 2006; García-Sanjuán et al., 2015; Lovén Wickman et al., 2016; Palant et al., 2015; Skrautvol & Nåden, 2015; Zhou et al., 2014). For example, one participant noted a shift to healthier alternatives such as choosing salads over greasy, fried foods (Jamieson et al., 2007). Changes in cooking methods were observed, with preferences noted for steaming and grilling over frying (García-Sanjuán et al., 2015). Moreover, food avoidance strategies enhanced the ability of these participants to adapt to work-related challenges (Fletcher & Schneider, 2006; Palant et al., 2015). One participant shared their practice of abstaining from eating during work hours to minimize discomfort, despite acknowledging this strategy as unconventional (Palant et al., 2015). Over time, the participants reflected on their dietary experiences and gradually recognized and accepted the influence of diet on their condition, leading to a more selective and refined approach to choosing foods (Czuber-Dochan et al., 2020; García-Sanjuán et al., 2015; Jamieson et al., 2007; Matinez-Riera et al., 2022; Nowlin et al., 2021; Palant et al., 2015; Rines et al., 2022; Rogers et al., 2021; Zhou et al., 2014). One participant described these dietary changes as an enlightening journey that had begun with confusion and experimentation and had ended with a personalized diet for effective symptom management (Matinez-Riera et al., 2022).

### Category 2. Disruption of Life and Mood

#### Theme 4. Reshaping Life Planning and Increasing Life Stress

This theme explores how food avoidance evolves as IBD becomes a central aspect in the life planning of patients and gradually increases their life stress. The original findings are (a) food-oriented life and (b) increased life stress.

Due to food avoidance, the patients were compelled to prioritize dietary considerations in organizing their work and daily life (Czuber-Dochan et al., 2020; Fletcher et al., 2008; Fletcher & Schneider, 2006; García-Sanjuán et al., 2015; Lovén Wickman et al., 2016; Matinez-Riera et al., 2022; Palant et al., 2015; Rines et al., 2022; Rogers et al., 2021; Scott et al., 2021; Sykes et al., 2015). For example, one patient highlighted the challenges of traveling and attending social functions, where food is central (Rogers et al., 2021). Moreover, most of the participants reported experiencing greater life stress attributable to their dietary limitations (Czuber-Dochan et al., 2020; Fletcher & Schneider, 2006; García-Sanjuán et al., 2015; Jamieson et al., 2007; Matinez-Riera et al., 2022; Palant et al., 2015; Rines et al., 2022; Rogers et al., 2021; Zhou et al., 2014). This stress often manifested in the need to prepare their own separate meals. One participant described cooking different meals to accommodate both their food avoidance needs and the food preferences of their spouse (Palant et al., 2015). Furthermore, the constant vigilance required in food selection was described as a source of stress (Zhou et al., 2014).

#### Theme 5: Negative Emotional Challenges

This theme addresses the array of negative emotions patients with IBD experience due to food avoidance. The original findings are (a) despondency linked to dietary changes, (b) confusion over dietary choices, (c) fear of food following adverse experiences, (d) concerns about malnutrition, (e) stigmatization related to food avoidance, and (f) distress associated with abstaining from favorite foods.

Patients often adjust their diet in hopes of improving their condition, although emotional setbacks are frequent. Many feel dispirited by the monotony of their “safe” diet and their unsuccessful avoidance attempts (Fletcher et al., 2008; Fletcher & Schneider, 2006; Jamieson et al., 2007; Palant et al., 2015; Rines et al., 2022; Zhou et al., 2014). When symptoms did not improve, the lengthy trial-and-error process required to find suitable foods became a source of discouragement (Zhou et al., 2014). Meanwhile, patients were confused by symptoms returning even after food avoidance (Czuber-Dochan et al., 2020; García-Sanjuán et al., 2015; Rines et al., 2022; Sykes et al., 2015; Zhou et al., 2014). One participant described the difficulties faced in continually changing their list of foods to avoid (Sykes et al., 2015). In addition, the fear of experiencing post-consumption symptoms led to an aversion to eating (Czuber-Dochan et al., 2020). Concerns over malnutrition were prevalent, with patients required to think about how to maintain their weight in light of the limited number of food choices available (Chen et al., 2017; Czuber-Dochan et al., 2020; Palant et al., 2015). Shame from food avoidance was commonly reported as arising during social situations (Matinez-Riera et al., 2022; Palant et al., 2015; Rines et al., 2022; Rogers et al., 2021). The participants reduced their outings as a strategy to cope with the embarrassment of food avoidance in social settings (Matinez-Riera et al., 2022). Furthermore, many expressed deep emotional distress when avoiding their favorite foods (Czuber-Dochan et al., 2020; Fletcher & Schneider, 2006; Palant et al., 2015; Rines et al., 2022). These emotions were identified as complex and interconnected.

### Category 3: Needs and Expectations

#### Theme 6: The Role of Family and Friends in Dietary Management

This theme highlights the significance of support received from family and friends in improving food avoidance strategies and the desire of patients for assistance. The original findings are (a) the positive influence of family environment on food avoidance management and (b) the yearning of patients for caring and understanding.

The research results in the included studies indicate that family members play a crucial role in improving the success of patient dietary adjustments. In one of the studies, family encouragement helped a child with IBD eat healthier, demonstrating the importance of caregivers in promoting healthy eating habits (Chuong et al., 2019). In cases where the patient with IBD was a parent, they consciously strived to manage their food avoidance in ways that promoted a positive food relationship for their children (Czuber-Dochan et al., 2020). In addition, the participants expressed a strong desire to receive care and understanding from friends and family (Matinez-Riera et al., 2022).

#### Theme 7. Workplace Support Deficiencies

This theme focuses on the challenges patients with IBD face due to the inadequate support received in their work environments. The original findings are (a) insufficient collegial support for food avoidance and (b) conflicts between work settings and eating habits.

The participants in the included studies frequently reported needing more understanding and support from colleagues with regard to their food avoidance (Czuber-Dochan et al., 2020; Matinez-Riera et al., 2022; Zhou et al., 2014). For example, one reported feeling alienated during social gatherings at work due to colleagues having difficulty understanding their condition (Matinez-Riera et al., 2022). In addition, work environments were reported to clash frequently with participants' dietary requirements (Matinez-Riera et al., 2022; Zhou et al., 2014). One described experiencing difficulties in avoiding spicy foods in a workplace where spicy foods were prevalent (Zhou et al., 2014). Another challenge noted by one participant with Crohn’s disease was the clash between the individual need for slower eating and digestion and the constraints imposed by short meal break times (Matinez-Riera et al., 2022).

#### Theme 8: Lack of Professional Dietary Guidelines

This theme addresses the obstacles patients encounter in obtaining professional dietary guidance for IBD. The original findings are (a) limited physician knowledge about IBD-specific diets, (b) the inadequate attention paid by medical staff to patient diets, (c) conflicting dietary instructions, and (d) the limited number of dietitians specializing in IBD.

A common perception among the participants in the included studies was that doctors lacked expertise in IBD-related nutrition (Barriteau Siiri et al., 2022; García-Sanjuán et al., 2015; Matinez-Riera et al., 2022; Nowlin et al., 2021; Rogers et al., 2021; Skrautvol & Nåden, 2015). One was frustrated by their doctor’s lack of dietary knowledge and interest (Barriteau Siiri et al., 2022). Some attributed their doctors’ indifference to providing dietary advice to a general skepticism about the impact of diet on IBD (Barriteau Siiri et al., 2022; Fletcher et al., 2008; Jamieson et al., 2007; Matinez-Riera et al., 2022; Palant et al., 2015; Rines et al., 2022; Rogers et al., 2021). Furthermore, the participants were frequently given conflicting dietary recommendations (Czuber-Dochan et al., 2020; Zhou et al., 2014). One noted discrepancies between advice given by traditional Chinese and Western medicine doctors (Zhou et al., 2014). The participants perceived that “healthy diets” prescribed for the general public are wrong for them (Czuber-Dochan et al., 2020). In addition, the scarcity of dietitians with IBD expertise was highlighted (García-Sanjuán et al., 2015; Matinez-Riera et al., 2022; Skrautvol & Nåden, 2015), with the participants reporting receiving inadequate dietary support from registered dietitians (Barriteau Siiri et al., 2022).

### Category 4: Social Alienation

#### Theme 9: Alienation from Intimacy

This theme addresses the alienation that patients with IBD experience from family and friends due to food avoidance. The original findings are (a) tension in peer relationships and (b) estrangement from family activities.

The participants reported their food avoidance resulting in lower social interactivity that increased misunderstandings with friends (Rines et al., 2022; Rogers et al., 2021). For example, one patient felt perceived as aloof for declining to attend food-centric social gatherings (Rogers et al., 2021). Moreover, food avoidance was shown to isolate these individuals from their families (Alexakis et al., 2015; Czuber-Dochan et al., 2020), exemplified by one participant who felt excluded due to different dietary needs and the extra effort required from their parents (Alexakis et al., 2015).

#### Theme 10: Alienation of Culinary Culture

This theme explores how food avoidance due to IBD leads to alienation from traditional culinary practices. The original findings are (a) avoidance of traditional ethnic foods and (b) conflict with dietary norms and cultural practices.

The participants frequently abstained from consuming culturally significant foods, representing a significant shift in their dietary habits (Alexakis et al., 2015; Scott et al., 2021; Sykes et al., 2015; Zhou et al., 2014). The narrative of one participant captures the initial struggle and eventual adaptation made to avoid traditional soul food (Scott et al., 2021). This avoidance affects personal habits and leads to a broader cultural disconnection (Alexakis et al., 2015; Scott et al., 2021). One participant highlighted the integral nature of food to their ethnic culture, underscoring the challenge of navigating social events centered around food, such as weddings and family gatherings (Alexakis et al., 2015).

#### Theme 11: Social Distancing

This theme describes the social isolation experienced by patients with IBD as a result of their food avoidance. The original findings are (a) decreased frequency of social outings and (b) diminished engagement in social activities.

Many of the participants reported that food avoidance led to less frequent participation in outings (Alexakis et al., 2015; Czuber-Dochan et al., 2020; Fletcher & Schneider, 2006; Palant et al., 2015; Rogers et al., 2021; Scott et al., 2021; Sykes et al., 2015). One example of this challenge highlighted the difficulties faced in dining out due to IBD-related restrictions (Palant et al., 2015). In addition, the participants reported being unable to immerse themselves in social activities, even when they joined in, due to food avoidance (Alexakis et al., 2015; Czuber-Dochan et al., 2020; Fletcher & Schneider, 2006; García-Sanjuán et al., 2015; Lovén Wickman et al., 2016; Palant et al., 2015; Rines et al., 2022; Sykes et al., 2015). For example, one participant described feeling detached from the overall experience of their wedding, unable to partake in celebratory eating, and limited to asking others to describe the food (Rines et al., 2022).

## Discussion

### Introduction to Management Challenges in Food Avoidance

The synthesized data from this study underscores the significant time and energy patients with IBD invest in dietary decision-making due to their unique food-avoidance needs. The lack of professional dietary advice often leads these patients to self-manage their diet, resulting in a spectrum of emotional responses, including confusion, fear, worry, stigma, and devastation. This food-avoidance approach makes maintaining intimate, social, and cultural connections difficult, which in turn negatively impacts the individual’s professional life. Despite these difficulties, the participants in these studies demonstrated resilience by adapting their diets and improving their handling of work and life demands. This resilience aligns with psychological theories emphasizing positive adaptation in adversity (Luo et al., 2018). The findings emphasize the importance of health care providers providing specific, clear, and practical nutritional recommendations and addressing the emotional and social issues of these patients when they arise. Improving support from family, friends, and health care providers is critical to increasing psychological resilience in these patients and enabling their better management of dietary difficulties.

### Enhancing System Support for Effective Dietary Management

Our study highlights a significant need for external understanding and support in managing the IBD diet, with a notable current lack of support from workplaces and medical professionals. Support from family and friends is crucial to improving food avoidance practices, particularly in adolescents. A strong support system helps reduce food avoidance in patients with IBD. First, expert medical support is essential to managing IBD comprehensively. Echoing the findings of Crooks et al. (2022), the participants in the included studies often lacked adequate dietary guidance from medical staff and dietitians. However, a significant driver of this problem may be patient skepticism toward the dietary advice provided by health care professionals (Czuber-Dochan et al., 2020; Rines et al., 2022). Moreover, dietitians were shown to frequently fail to tailor dietary recommendations to the specific needs and disease status of patients with IBD, leading to inconsistent advice across different practitioners (Palant et al., 2015; Rogers et al., 2021). Second, the inadequate disease-related knowledge of family, friends, and colleagues was shown to amplify the dietary challenges faced by these patients. Insufficient knowledge influences dietary choices, exacerbates negative emotions, and increases food avoidance. IBD dietary management training and other relevant measures must be implemented to enhance health care practitioner expertise on IBD dietary needs. Regular IBD care programs should also incorporate personalized dietary management. Ultimately, these patients must gain a deeper understanding of their diet and garner support from their kin, acquaintances, and beyond, thereby reducing food avoidance.

### Inconsistent Views on Food Avoidance

Two main contradictions in the 11 IBD food avoidance topics were identified in this study. First, diet is crucial to IBD management. Food avoidance can help patients manage symptoms and eat healthier. However, excessive avoidance often results in excluding beneficial foods, particularly during remission, which creates a monotonous diet (Marsh et al., 2019). Differences in the impact of food avoidance may stem from individual approaches to dietary modification that are not professionally advised. Second, while patients with IBD often avoid food, excessive avoidance can harm mood and life quality. The European Society for Clinical Nutrition and Metabolism clarified that no specific “IBD diet” is recommended for inducing or maintaining remission (Bischoff et al., 2023), and Fitzpatrick underscored the importance of identifying maladaptive eating patterns in IBD dietary management (Fitzpatrick et al., 2022). Therefore, health care practitioners must ensure patients do not avoid healthy foods and periodically examine/adjust their dietary management practices based on their current nutritional and illness status. Furthermore, clearer dietary management guidelines and evidence-based recommendations are necessary to optimize the balance between symptom control and nutritional adequacy. Future nutritional evaluation tools for patients with IBD may help screen and treat food avoidance.

### Strengths

This study’s strengths include the systematic search strategy used and the inclusion of 19 original qualitative studies on the experiences and perceptions of patients with IBD regarding food avoidance. Six of these studies were rated grade A and 13 were rated Grade B, reflecting a high overall level of quality. Moreover, this study’s inclusivity and analytical depth, driven by its systematic review of food avoidance literature and inclusion of patients with IBD across diverse ages and disease stages, enhance its contribution to understanding food avoidance experiences. Thus, this study is relatively more helpful in elucidating the full range of food avoidance experiences and points of view of patients with IBD.

### Limitations

First, most of the studies examined in this study address food avoidance in terms of patient self-reported dietary experiences, which may have contributed to the insufficient findings regarding the causes of food avoidance. Future investigations may specifically investigate factors influencing food avoidance. Second, excluding patients who received nasogastric feeding limited the generalizability of the food avoidance experiences of patients with IBD. Future research should consider the food avoidance experiences of naso-fed patients to better understand and support their dietary transition from total enteral nutrition to regular diets.

### Conclusions

This study provides a deeper and more comprehensive evaluation of the authentic experiences and feelings of patients with IBD regarding food avoidance using a meta-synthesis of 19 qualitative research articles. Valuable insights are provided to guide these patients in improving their diet management. Also, the conflicts and complexities patients encounter during the food avoidance process are depicted, highlighting the difficulties patients with this condition face in managing their diet. Patients expect to improve their disease status through food avoidance, hoping for positive outcomes. However, they gradually alienate themselves from the outside world during the process, disrupting their original normal life and exacerbating negative emotions. Therefore, these patients must be actively guided to adopt correct attitudes toward dietary management and provided psychological counseling. Meanwhile, social support influences the food avoidance of patients with IBD over their disease management journey. Further research into food avoidance is vital, with important issues in need of clarification including the determinants of avoidance behavior, personalized dietary plans, how to better assist patients in managing their diet and symptoms effectively, and how to foster robust social support networks for patients with IBD.
